# Effectiveness of mechanical thrombectomy in cancer-related stroke and associated factors with unfavorable outcome

**DOI:** 10.1186/s12883-021-02086-y

**Published:** 2021-02-06

**Authors:** Eung-Joon Lee, Jeonghoon Bae, Hae-Bong Jeong, Eun Ji Lee, Han-Yeong Jeong, Byung-Woo Yoon

**Affiliations:** 1 Department of Neurology, Seoul National University Hospital, 101 Daehak-ro, Jongno-gu, Seoul, 03080 Republic of Korea; 2 Department of Neurology, Chung-Ang University Hospital, Seoul, Republic of Korea; 3 Department of Radiology, Soonchunhyang University Seoul Hospital, Seoul, Republic of Korea

**Keywords:** Stroke, Endovascular treatment, Neoplasm, Neoplasm metastasis, Outcome

## Abstract

**Background:**

The effectiveness of mechanical thrombectomy (MT) in cancer-related stroke (CRS) is largely unknown. This study aims to investigate the clinical and radiological outcomes of MT in CRS patients. We also explored the factors that independently affect functional outcomes of patients with CRS after MT.

**Methods:**

We retrospectively reviewed 341 patients who underwent MT after acute ischemic stroke onset between May 2014 and May 2020. We classified the patients into CRS (*n* = 34) and control (*n* = 307) groups and compared their clinical details. Among CRS patients, we analyzed the groups with and without good outcomes (3-months modified Rankin scale [mRS] score 0, 1, 2). Multivariate analysis was performed to investigate the independent predictors of unfavorable outcomes in patients with CRS after MT.

**Results:**

A total of 341 acute ischemic stroke patients received MT, of whom 34 (9.9%) had CRS. Although the baseline National institute of health stroke scale (NIHSS) score and the rate of successful recanalization was not significantly different between CRS patients and control group, CRS patients showed more any cerebral hemorrhage after MT (41.2% vs. controls 23.8%, *p* = 0.037) and unfavorable functional outcome at 3 months (CRS patients median 3-month mRS score 4, interquartile range [IQR] 2 to 5.25 vs. controls median 3-month mRS score 3, IQR 1 to 4, [*p* = 0.026]). In the patients with CRS, elevated serum D-dimer level and higher baseline NIHSS score were independently associated with unfavorable functional outcome at 3 months (adjusted odds ratio [aOR]: 1.524, 95% confidence interval [CI]: 1.043–2.226; aOR: 1.264, 95% CI: 1.010–1.582, respectively).

**Conclusions:**

MT is an appropriate therapeutic treatment for revascularization in CRS patients. However, elevated serum D-dimer levels and higher baseline NIHSS scores were independent predictors of unfavorable outcome. Further research is warranted to evaluate the significance of these predictors.

## Introduction

Cerebrovascular disease affects around 15% of all patients with malignancy throughout their life. Patients with cancer frequently experience acute ischemic stroke (AIS) [1]. The pathophysiology of AIS in cancer is vary. Among these, cancer-related stroke (CRS) by intravascular coagulopathy by tumor-cell derived cytokines or microparticles, is of particular concern [2, 3]. Serum D-dimer level is often high (> 1.11 μg/mL) and multiple lesions in various vascular territories are associated with CRS [4, 5]. With the advancement of anti-cancer therapy, the survival period has been prolonged; therefore, CRS and the need for thrombolysis has increased [6]. However, due to conditions such as thrombocytopenia, or recent surgery, patients with CRS are often excluded for intravenous recombinant tissue plasminogen activator (rtPA) administration [7–9]. Therefore, mechanical thrombectomy (MT) for occluded large vessels can be a particularly important treatment option for CRS patients [10, 11]. However, although there have been earlier studies showing the overall outcomes of MT in patients with CRS [12–17], the effectiveness of MT on CRS remains controversial as patients with active cancer were excluded from previous important clinical trials [18–21]. To the best of our knowledge, there have not been any studies investigating the independent factors associated with the functional outcomes of CRS after MT. Therefore, this study aimed to investigate the effectiveness of MT in CRS patients. We also explored the factors that were independently associated with unfavorable functional outcomes.

## Methods

### Subjects

We retrospectively reviewed the medical records of all 372 consecutive patients with AIS who received MT in the Seoul National University Hospital (SNUH) between May 2014 and May 2020. The following patients were excluded: MT performed > 24 h after onset; patients with active hematologic malignancies, intracranial neoplasm, or central nervous system metastasis; and patients whose clinical follow-up with a modified Rankin scale (mRS) score at 3-month was unavailable. The final study population was 341. Patients who received MT were divided into two groups: the CRS group and the control. CRS was defined as a cryptogenic ischemic stroke occurring in active cancer patients who had elevated D-dimer levels and/or multiple cerebral territory involvement on diffusion weighted imaging. Active cancer patients referred to those who were diagnosed and currently undergoing or refusing treatment for malignancy. Patients diagnosed during hospitalization for stroke were also included. The involvement of multiple cerebral territories was defined as the presence of multiple ischemic lesions in the unilateral anterior and posterior circulation, bilateral anterior circulation, or bilateral anterior and posterior circulation [22].

This study was approved by the institutional review board (IRB number: 1009–062-332) of SNUH. Informed consent was waived by the IRB due to the retrospective nature of the study.

### Clinical assessment

Age, sex, pre-stroke mRS scores, baseline National Institute of Health Stroke Scale (NIHSS) scores, conventional vascular risk factors (history of hypertension, diabetes mellitus, dyslipidemia, atrial fibrillation, and previous ischemic stroke or transient ischemia attack, coronary artery disease, and smoking status), laboratory findings (hemoglobin level, White blood cells [WBC] count, platelet counts, prothrombin time, total cholesterol level, level of high-density lipoprotein cholesterol [HDL-C], level of low-density lipoprotein cholesterol [LDL-C], and level of C-reactive protein [CRP]), whether the stroke onset is in hospital, occlusion site where the MT was performed, and the status of receiving intravenous rtPA administration of each subject were collected from the database of SNUH’s stroke registry. The occluded location was classified into M1 and M2 of middle cerebral artery (MCA), internal carotid artery (ICA), and posterior circulation (posterior cerebral artery [PCA] and basilar artery [BA]). Regarding the time variable associated with MT, onset-to-puncture time and puncture-to-reperfusion time were gathered. When the onset time was unclear, the last normal time was used as the reference and the reperfusion time was defined as the first reperfusion with mTICI ≥2a.

The decision to perform MT was determined based on multimodal computed tomography (CT) such as non-contrast CT, CT angiography, and perfusion CT. Magnetic resonance image (MRI) was additionally used in some cases. Intravenous rtPA was used within 4.5 h after symptom onset if the patients met the relevant criteria. MT was performed using stent retrievers or a forced suction system selected, depending on the preference of the neurointerventionalists. Unless there was a specific contraindication, all patients underwent an MRI during hospitalization and patients were classified according to the Trial of ORG 10,172 in Acute Stroke Treatment (TOAST) criteria [23]. The choice of antithrombotic agents after MT was decided according to individual cases.

In the CRS group, we additionally collected the location and stage of cancer, presence of systemic metastasis, pathologic type whether adenocarcinoma or not, and serum D-dimer levels. Regarding the indicators of clinical outcomes, mRS score at discharge, NIHSS score at discharge, mRS score at 3 months, mortality during hospitalization and after 3 months, and causes of mortality at 3 months were gathered. We defined unfavorable outcome as a 3-month mRS score ≥ 3 [24].

### Radiologic assessment

A single neurologist and a single radiologist, blinded to the clinical information, independently analyzed the angiographic data; the recanalization status was evaluated according to the modified thrombolysis in cerebral infarction (mTICI) scale through digital subtraction angiography [25]. Successful recanalization was defined as mTICI 2b or 3.

Cerebral hemorrhage included any intracerebral or subarachnoid hemorrhage found on the 24 h follow-up imaging. All patients who received MT were followed up immediately after 24 h by CT or MRI according to the stroke management protocol of SNUH. Reported hemorrhages were categorized according to the Heidelberg Bleeding Classification (HBC) [26].

### Statistical analysis

Statistical analysis was performed using IBM SPSS version 25 (IBM Corp., Armonk, NY, USA). Data were presented as means and medians with corresponding standard deviations and interquartile ranges, respectively. Univariate analyses (independent sample t test or Mann-Whitney U test for continuous variables, and χ2 test or Fisher’s exact test for categorical variables) were performed to compare demographics, vascular risk factors, laboratory findings, location of stroke onset, use of rtPA, and occlusion site between groups. We also compared radiological results such as reperfusion status and presence of any cerebral hemorrhage. Multivariate models were constructed to investigate what factors independently predicted unfavorable outcomes in the CRS group after MT. A two-sided *P* value of < 0.05 was considered statistically significant.

## Results

### Baseline characteristics of the total study participants

A total of 341 participated in our study. Thirty-four (9.9%) had CRS, while 307 (90.1%) had ischemic stroke due to other mechanisms. The mean age of the patients was 68.46 ± 13.2 years. Among the total 341 patients, 178 were male (52.2%). Age, sex, premorbid mRS scores, vascular disease risk factors and baseline NIHSS scores were not significantly different between the CRS and control group, except for history of atrial fibrillation (0% vs. 57.0%, *p* < 0.001). The levels of hemoglobin and platelet count were significantly lower (10.8 ± 2.1 g/dℓ vs. controls 13.1 ± 2.1 g/dℓ, 187.7 ± 105.9 × 10^3^/μL vs. controls 216.3 ± 72.3 × 10^3^/μL, *p* < 0.001, *p* < 0.001, respectively). However, leukocyte count and CRP levels were significantly higher (10.7 ± 6.3 × 10^3^/μL vs. controls 8.8 ± 3.5 × 10^3^/μL, 6.3 ± 8.2 mg/dℓ vs. controls 1.5 ± 3.5 mg/dℓ, *p* = 0.042, *p* < 0.001, respectively) in the CRS group. Furthermore, in-hospital stroke was significantly more frequent in the CRS group (32.4% vs. controls 7.8%, p < 0.001). There was no significant difference in the level of total cholesterol, triglyceride, HDL-C, LDL-C, occlusion site where MT was performed, and status of receiving intravenous rtPA between the two groups (Table 1).

**Table 1:** Baseline characteristics of the total participants

	Cancer-related stroke	Control	*p* value
	*n* (%) or mean ± SD or median (IQR)	*n* (%) or mean ± SD or median (IQR)	
Total	34 (100%)	307 (100%)	
Age, years	64.5 ± 11.4	68.9 ± 14.0	0.124
Sex, male [n (%)]	17 (50%)	161 (52.4%)	0.921
Premorbid mRS score	0 (0–1.75)	0 (0–0)	0.294
Baseline NIHSS score	18 (11–23)	15 (8–19)	0.290
Comorbidities			
Hypertension	22 (64.7%)	219 (71.3%)	0.768
Diabetes	14 (41.1%)	106 (34.5%)	0.621
Hyperlipidemia	9 (26.5%)	139 (45.3%)	0.328
Coronary artery disease	5 (14.7%)	59 (19.2%)	0.142
Prior stroke or TIA	6 (17.7%)	82 (26.7%)	0.306
Smoking	5 (14.7%)	92 (30.0%)	0.072
Atrial fibrillation	0 (0%)	175 (57.0%)	< 0.001
Previous use of antithrombotics†	13 (38.2%)	98 (31.9%)	0.336
Laboratory findings			
Hemoglobin, g/dℓ	10.8 ± 2.1	13.1 ± 2.1	< 0.001
White blood cells, 10^3^/μL	10.7 ± 6.3	8.8 ± 3.5	0.042
Platelet counts, 10^3^/μL	187.7 ± 105.9	216.3 ± 72.3	< 0.001
Prothrombin time, sec/INR	1.2 ± 0.2	1.1 ± 0.3	0.562
Total cholesterol, mg/dℓ	150.2 ± 47	160.2 ± 42.3	0.134
Triglyceride, mg/dℓ	110.4 ± 52.7	99.0 ± 53.5	0.172
HDL cholesterol, mg/dℓ	38.1 ± 17	47.2 ± 15.1	0.094
LDL cholesterol, mg/dℓ	92.1 ± 32	97.5 ± 37.5	0.328
C-reactive protein, mg/dℓ	6.3 ± 8.2	1.5 ± 3.5	< 0.001
In-hospital Stroke	11 (32.4%)	24 (7.8%)	< 0.001
Occlusion site			
Middle cerebral artery	24 (70.6%)	172 (56.0%)	0.143
M1	18 (52.9%)	118 (38.4%)	0.139
M2	6 (17.6%)	54 (17.6%)	0.993
Anterior cerebral artery	1 (2.9%)	16 (5.2%)	0.478
Posterior cerebral artery/Basilar artery	4 (11.8%)	54 (17.6%)	0.479
Internal carotid artery	5 (14.7%)	63 (20.5%)	0.504
Common carotid artery	0 (0%)	2 (0.6%)	0.998
Use of intravenous rtPA	9 (26.5%)	80 (26.1%)	0.959

*Abbreviations*: *SD* standard deviation, *IQR* interquartile range, *mRS* modified Rankin scale, *NIHSS* National institute of health stroke scale, *TIA* transient ischemic attack, *INR* international normalized ratio, *HDL* High-density lipoprotein, *LDL* low-density lipoprotein, *rtPA* recombinant tissue plasminogen activator

†Antiplatelets and anticoagulation agents

### Comparison of the outcomes of MT between CRS and control groups

There was no statistically significant difference in time variables such as onset-to-puncture time and puncture-to-reperfusion time between two groups. The rate of successful recanalization was not significantly different (76.5% vs. controls 87.6%, *p* = 0.103) yet any cerebral hemorrhage was significantly more frequent in the CRS group (41.2% vs. controls 23.8%, *p* = 0.037). Both, immediate clinical outcomes as well as short-term outcomes were all significantly unfavorable in the CRS group (median CRS group mRS score at discharge 5, interquartile range (IQR) 3 to 5, controls median mRS at discharge 3, IQR 1 to 5, *p* = 0.044; median CRS group mRS score at 3 months 4, IQR 2 to 5.25, controls median mRS at 3 month 3, IQR 1 to 4, *p* = 0.026). Mortality during the hospitalization and at 3 months was significantly higher (20.6% vs. controls 5.9%, *p* = 0.009 and 26.5% vs. controls 6.8%, *p* < 0.001, respectively) in the CRS group. However, most patients died because of the stroke itself and the causes of mortality at 3 months were not significantly different between two groups. (Table 2).

**Table 2:** The results after mechanical thrombectomy of cancer-related stroke and controls

	Cancer-related stroke	Controls	*p* value
	*n* (%) or mean ± SD or median (IQR)	*n* (%) or mean ± SD or median (IQR)	
Total	34 (100%)	307 (100%)	
Time variable			
Onset-to-puncture time, min	327.5 (170.25–507.5)	330 (185–602.5)	0.751
Puncture-to-reperfusion time, min	66.5 (42.5–90.5)	60 (41–90)	0.784
Radiological outcomes			
Reperfusion status			0.758
mTICI 0, 1	2 (5.9%)	15 (4.9%)	
mTICI 2a	5 (14.8%)	23 (7.5%)	
mTICI 2b	14 (41.2%)	112 (36.5%)	
mTICI 3	13 (38.2%)	157 (51.1%)	
Successful recanalization	26 (76.5%)	269 (87.6%)	0.103
Any cerebral hemorrhage	14 (41.2%)	73 (23.8%)	0.037
HBC Class 1a	0	4	
HBC Class 1b	2	21	
HBC Class 1c	3	18	
HBC Class 2	7	25	
HBC Class 3a	0	0	
HBC Class 3b	0	0	
HBC Class 3c	2	5	
HBC Class 3d	0	0	
Craniectomy	3 (21.4%)	18 (24.6%)	0.452
Clinical outcomes			
NIHSS score at discharge	13 (8–30)	5 (2–12.5)	< 0.001
mRS score at discharge	5 (3–5)	3 (1–5)	0.044
mRS score at 3 months	4 (2–5.25)	3 (1–4)	0.026
Mortality during the hospitalization	7 (20.6%)	18 (5.9%)	0.009
Mortality at 3 months	9 (26.5%)	21 (6.8%)	< 0.001
Causes of death at 3 months			0.687
Stroke	7	15	
Sepsis	1	3	
Progression of cancer	1	0	
Others	0	3	

*Abbreviations*: *SD* standard deviation, *IQR* interquartile range, *min* minutes, *mTICI* modified thrombolysis in cerebral infarction, *HBC* the Heidelberg Bleeding Classification, *mRS* modified Rankin Scale, *NIHSS* National institute of health stroke scale

### Baseline characteristics and outcomes according to functional outcomes at 3 months after stroke of CRS patients

Among a total of 34 patients, only 11 (32.4%) subjects showed good functional outcome (mRS scores 0–2) at 3 months. In this group, the proportion of men was significantly higher (81.8% vs. controls 34.7%, *p* = 0.026) and exhibited significantly less stroke severity (median baseline NIHSS score 9, IQR 7.5 to 13.5 vs. controls median baseline NIHSS score 20, IQR 17 to 25, *p* < 0.001). Vascular risk factors, previous use of antithrombotic agents, status of receiving rtPA and occluded site were not significantly different between two groups. Onset-to-puncture time, puncture-to-reperfusion time and the rate of successful recanalization rate were not significantly different. However, the group with favorable outcome showed significantly less any cerebral hemorrhage after MT (9.1% vs. unfavorable group 60.9%, *p* < 0.001). There was no significant difference in cancer location and type between the two groups. However, the cancer stage was more aggravated, the systemic metastasis was more prevalent (78.3% vs. favorable group 36.4%, *p* = 0.016) in the unfavorable group. Lastly, serum D-dimer level were significantly elevated in the unfavorable group (Unfavorable group D-dimer level median 16.95 mg/dL, IQR 14.55 to 26.60 vs. favorable group D-dimer level median 10.7 mg/dL, IQR 6.66 to 17.71 mg/dL, *p* = 0.028, see Table 3).

**Table 3:** Baseline characteristics and outcomes according to functional outcomes at 3 months of cancer-related stroke

Characteristics	Favorable outcome	Unfavorable outcome	*p* value
	3-Month mRS score 0–2	3-Month mRS score 3–6	
Total	*N* = 11	*N* = 23	
Age, year, median (IQR)	70 (57–73.50)	65 (59–72.50)	0.690
Male	9 (81.8%)	8 (34.7%)	0.026
Pre mRS score, median (IQR)	0 (0–0)	0 (0–2.5)	0.153
Initial NIHSS score, median (IQR)	9 (7.5–13.5)	20 (17–25)	< 0.001
Vascular risk factors			
Prior stroke or TIA	4 (36.3%)	2 (8.7%)	0.070
Coronary artery disease	1 (9.1%)	4 (17.4%)	0.507
Hypertension	7 (63.6%)	15 (65.2%)	0.928
Diabetes mellitus	4 (36.4%)	8 (34.8%)	0.928
Dyslipidemia	4 (36.4%)	9 (39.1%)	0.878
Smoking	3 (27.3%)	2 (8.7%)	0.300
Previous use of antithrombotics†	4 (30.8%)	9 (39.1%)	0.876
Administration of IV-rtPA	1 (9.1%)	8 (34.8%)	0.214
Occlusion site			0.861
Middle cerebral artery, M1	5 (45.4%)	13 (56.5%)	
Middle cerebral artery, M2	2 (18.2%)	4 (17.4%)	
Internal carotid artery	3 (27.3%)	2 (8.7%)	
Anterior cerebral artery	0 (0%)	1 (4.3%)	
Posterior cerebral artery/Basilar artery	1 (9.1%)	3 (13.1%)	
In-Hospital Stroke	4 (36.4%)	7 (30.4%)	0.731
Time variables			
Onset-to-puncture time, min, SD	471 (290.24)	364.39 (224.31)	0.612
Puncture-to-reperfusion time, min, SD	74.45 (27.48)	80.17 (46.13)	0.856
Radiologic Outcomes			
Recanalization status			0.160
mTICI 3, 2b	11 (100%)	16 (69.6%)	
mTICI 2a	0 (0%)	5 (21.7%)	
mTICI 1, 0	0 (0%)	2 (8.7%)	
Any cerebral hemorrhage	1 (9.1%)	13 (56.5%)	< 0.001
Cancer by location			0.510
Pancreatic cancer	2 (18.2%)	7 (30.4%)	
Lung cancer	4 (36.4%)	3 (13.0%)	
Bile duct cancer	2 (18.2%)	3 (13.0%)	
Liver cancer	1 (9.1%)	2 (8.7%)	
Gastric cancer	0 (0%)	2 (8.7%)	
Colon cancer	1 (9.1%)	2 (8.7%)	
Ovarian cancer	1 (9.1%)	2 (8.7%)	
Breast cancer	0 (0%)	2 (8.7%)	
Cancer stage			0.018
III	7 (63.6%)	5 (21.7%)	
IV	4 (36.4%)	18 (78.3%)	
Cancer type			0.280
Adenocarcinoma	11(100%)	19 (82.6%)	
Others	0 (0%)	4(17.4%)	
Systemic metastasis	4 (36.4%)	18 (78.3%)	0.016
D-dimer, median (IQR)	10.7 (6.66–17.71)	16.95 (14.55–26.60)	0.028

*Abbreviations*: *mRS* modified Rankin scale, *IQR* interquartile range, *NIHSS* National institute of health stroke scale, *TIA* transient ischemic attack, *IV* intravenous, *rtPA* recombinant tissue plasminogen activator, *mTICI* modified thrombolysis in cerebral infarction

†Antiplatelets and anticoagulation agents

### Factors associated with unfavorable outcome in CRS after MT

In a univariate analysis of CRS patients, the following factors were significantly associated with unfavorable outcomes: higher initial NIHSS score, male gender, higher serum D-dimer level, occlusion of the M2 segment of middle cerebral artery, presence of systemic metastasis and any cerebral hemorrhage after endovascular treatment. In a binary logistic regression analysis (adjusting for demographics and variables achieving a *p* value of < 0.1 in the univariate analyses), elevated serum D-dimer level and higher baseline NIHSS score independently predict unfavorable outcome for CRS patients after MT (adjusted odds ratio (aOR): 1.524, 95% confidence interval (CI): 1.043–2.226; aOR: 1.264, 95% CI: 1.010–1.582, respectively, see Table 4).

**Table 4:** Factors associated with unfavorable outcome (3-month mRS ≥ 3) in cancer-related stroke after mechanical thrombectomy

Covariate	Crude OR (95% Cl)	*p* value	Adjusted OR*(95% Cl)	*p* value
Initial NIHSS score	1.321 (1.104–1.580)	0.002	1.264 (1.010–1.582)	0.041
Pre-stroke mRS score	2.418 (0.916–6.388)	0.075	3.207 (0.293–35.134)	0.340
Prior stroke or TIA	0.167(0.025–1.115)	0.065	2.380 (0.007–14.578)	0.442
Sex (Men)	0.119 (0.020–0.686)	0.017	0.771 (0.004–5.983)	0.575
Occlusion of MCA, M2	0.167 (0.025–1.115)	0.065	0.077 (0.001–1.967)	0.442
Occlusion of ICA	0.080 (0.008–0.835)	0.035	0.557 (0.006–4.924)	0.798
D-Dimer	1.617 (1.159–2.255)	0.049	1.524 (1.043–2.226)	0.029
Any cerebral hemorrhage	15.556 (1.690–143.174)	0.015	6.676 (0.052–18.481)	0.443
Systemic metastasis	6.300 (1.300–30.533)	0.022	1.291(0.847–1.967)	0.118

* Adjusted for baseline NIHSS scores, Pre-stroke mRS, Prior history of stroke or TIA, Sex, Occlusion of M2 segment of MCA and ICA, presence of any cerebral hemorrhage and systemic metastasis

*Abbreviations*: *OR* odds ratio, *CI* confidence interval, *NIHSS* National institute of health stroke scale, *TIA* Transient ischemia attack, *mRS* modified Rankin scale, *MCA* Middle cererbral artery, *ICA* internal carotid artery

## Discussion

In this study, although the rate of successful recanalization was not significantly different between the CRS and control groups, the degree of functional recovery was significantly worse in CRS than that of the control group. These were similar to the findings of previous studies conducted on the MT results of patients with active cancer. Generally, recanalization rate was found to be relatively high, but the degree of short-term functional recovery was found to be poor, and a high mortality rate at 3 months was observed. (Table 5).

**Table 5:** Comparison of the results between present and earlier studies

Authors	Lee et al. [12]	Jung et al. [13]	Cho et al. [14]	Sallustio et al. [15]	Jeon et al. [16]	Oki et al. [17]	Present report
Number of cases	26	19	27	24	62	12	34
Age (years, mean/median)	63 (median)	69 (median)	69 (mean)	69 (mean)	70 (median)	64 (mean)	64.5 (mean)
Male, sex	18 (69.2%)	9 (47.4%)	20 (74.1%)	8 (33.3%)	29 (46.8%)	5 (41.7%)	17 (50%)
Baseline NIHSS (mean/median)	14 (median)	16 (median)	11 (median)	14.2 (mean)	15 (median)	17.2 (mean)	18 (median)
Cancer stage IV (%)	N/A	89.5%	29.6%	41.7%	69.4%	58.0%	64.7%
Prestroke mRS ≤ 2	N/A	N/A	100.0%	95.8%	N/A	83.0%	88.2%
IV-rtPA	19.2%	15.8%	63.0%	45.8%	14.5%	33.0%	26.5%
Punture-to-reperfusion time (min, mean/median)	59.5 (median)	30 (median)	N/A	52.9 (mean)	29 (median)	70 (mean)	66.5 (median)
Succesful reperfusion (mTICI 2b or 3)	88.5%	63.2%	85.2%	76.9%	75.8%	83.0%	76.5%
mRS 0–2 at 3 months	23.1%	15.8%	37.0%	41.7%	17.7%	25.0%	32.0%
Mortality at 3 months	30.8%	63.2%	33.3%	29.1%	48.4%	50.0%	26.5%
Mortality at 12 months	N/A	N/A	N/A	N/A	N/A	92.0%	N/A

*Abbreviations: NIHSS* National Institutes of Health Stroke Scale score, *rtPA* recombinant tissue plasminogen activator; *mTICI* modified thrombolysis in cerebral infarction, *mRS* modified Rankin scale, *N/A* not available

According to our analysis, most deaths within 3 months (77.8%) were related to cerebral infarction and occurred during hospitalization. Similar to our results, Lee et al. reported that most deaths are caused by the ischemic stroke itself rather than the progression of cancer [12]. After stroke, anti-cancer therapy was usually stopped until the stroke stabilized. Further, stroke affects mortality in patients with active cancer by various ways including the termination of malignancy treatment and the worsening of general conditions. Moreover, Oki et al. showed that 92% of patients with cancer-related stroke died within 1 y after the stroke occurred [17]. In cancer patients, thromboembolism and progression of cancer are major causes of death. Taking all these results into consideration, the cause of death within a short period after stroke in the CRS group is often the stroke itself, and the causes of long-term mortality might be the discontinuation of anti-cancer therapy after stroke and the general deconditioning. Therefore, it might be necessary to actively perform MT in a more selected group to achieve functional recovery in CRS, and to continue anti-cancer therapy. In addition, in some cases, MT in CRS could enable an improved quality of life. Further research on a larger scale of long-term prognosis of CRS after MT is needed.

In the comparison of baseline characteristics between the CRS group and the control, hemoglobin level was significantly lower and leukocyte count was significantly elevated in the CRS group. Inadequate hemoglobin level and elevated leukocyte count is frequently associated with cancer and cancer-related coagulopathy [27–29]. These factors are also associated with unfavorable prognosis after stroke [30]. Furthermore, CRP levels were significantly higher in the CRS group than the control group. As an inflammatory marker, higher CRP may reflect acute inflammation which trigger for thromboembolism, or CRP itself is related to coagulation pathway and therefore may activate the cancer-specific prothrombotic mechanisms [31, 32]. In previous studies, higher circulating levels of CRP are associated with unfavorable outcomes in patients with solid malignancies [33]. Distinctively, CRS is significantly more occurred during hospitalization. This may suggest the possibility that anti-cancer therapies such as chemotherapy and radiotherapy, and various invasive procedures may be related to pathogenesis of CRS [34]. Or, according to the reason for the patients’ hospitalization, infection or inflammation may brought about cancer-related coagulopathy. Further research about factors associated with CRS during hospitalization is needed.

Interestingly, even though there is no significant difference in the baseline NIHSS scores, rate of successful recanalization, time variables such as onset-to-puncture time and puncture-to-reperfusion time, and use of rtPA between CRS and control groups, any cerebral hemorrhage after MT was significantly higher in the CRS group. Whether post-procedural hemorrhage occur more frequently in active cancer patients is still debatable [35, 36]. However, in the case of cancer-related coagulopathy such as disseminated intravascular coagulation, activation of coagulation may lead to exhaustion of platelets and coagulation factors, and eventually bleeding [37]. Also, the platelet count of CRS group is significantly lower than control in our results. In addition, the risk of cerebral hemorrhage may have increased if liver function associated with synthesis of blood coagulation factors is affected by primary or metastatic cancer. Immediate and short-term clinical outcomes are all significantly unfavorable in CRS group than controls. This may be due to clinical course of cancer itself, or it could be explained by that it was because of cerebral hemorrhage after MT interrupted appropriate management of ischemic stroke.

In the CRS group, the location of cancer varied, however mainly pancreas, lung, and bile ducts. The type of cancer cell was overwhelmingly adenocarcinoma. All CRS patients had advanced-stage III or more. These findings are all consistent with the results of previous studies about CRS [34]. Bang. Et al identified that circulating cancer-cell extracellular vesicles were higher in lung adenocarcinoma and had a direct role in blood clotting [2]. Understanding the features and further research on the pathologic mechanism of CRS is necessary for development of its proper treatment.

Another notable finding of this study was that elevated serum D-dimer level and higher baseline NIHSS scores independently associated with unfavorable outcomes in CRS after MT. As nonspecific biomarker of hypercoagulability, high D-dimer levels have been known to be related to poor prognosis in CRS [38, 39]. They are also associated with disturbing recanalization and increasing reperfusion injury [40]. Thus, elevated D-dimer levels may also explain the high incidence of intracerebral hemorrhage after MT in CRS. In previous studies, baseline NIHSS scores have been shown to be associated with post-MT prognosis in patients with AIS [41–43]. Therefore, thrombectomy for CRS patients, especially in a case of patients with elevated serum D-dimer level and more severe stroke, needs more judicious decision and great care should be taken to prevent post-procedural cerebral hemorrhage.

The strength of this study is that we analyzed the independently associated factors of unfavorable outcome in patients with CRS after MT. To the best of our knowledge, this is the first study that investigates the predictors of functional outcome within CRS patients after MT. As an emerging subtype of ischemic stroke, CRS is associated with cancer-related coagulopathy and its rates are expected to increase in future. This will encourage further research to take place to better understand and treat the disease.

This study has several limitations. First, it is a retrospective study with a relatively small number of patients from a single center. Larger sample study is needed to elucidate the effectiveness of MT in patients with CRS. Second, a detailed analysis of treatment status of cancer was not sufficiently performed. It was difficult to incorporate this due to the small sample size. Hence, only the location, stage, presence of metastasis and pathologic features of adenocarcinoma were included. Third, among the control group, patients with inactive cancer were not excluded. The effect that inactive cancer might have on stroke occurrence and prognosis is not clear. However, cancer-related coagulopathy is known to have a strong correlation with cancer activity, and the histological features of thrombi are also known to be different between stroke patients with active cancer and inactive cancer [44]. Therefore, we believe that there is little possibility that inactive cancer affected the occurrence and prognosis of the CRS that we focused on. Lastly, although there was no significant difference in the rate of MT performed in the M2 segment between CRS and control groups, it is likely that MT was less frequently performed in the CRS group than in the control group. Due to the medical condition of CRS patients, there are cases in which MT cannot be performed in consideration of risks and benefits. However, this study lacks consideration for these cases, including only the cases where MT was performed. These limitations should be considered while interpreting the study results.

## Conclusion

In conclusion, successful recanalization rate was similar in both CRS and control groups with no differences in baseline NIHSS scores and time variables. Thus, for revascularization, MT is an appropriate therapeutic treatment for CRS. However, post procedural intracerebral hemorrhage were more frequent and overall functional outcomes are unfavorable in the CRS group. Careful consideration of the D-dimer level and the baseline stroke severity is necessary when considering MT for patients with CRS. Further research is warranted to evaluate the significance of these predictors.

## Data Availability

Available upon reasonable request to the corresponding author

## Abbreviations

MT: Mechanical thrombectomy
CRS: Cancer-related stroke
AIS: Acute ischemic stroke
NIHSS: National institute of health stroke scale
aOR: Adjusted odds ratio
CI: Confidence interval
HBC: The Heidelberg bleeding classification
rtPA: Recombinant tissue plasminogen activator
WBC: White blood cells
HDL-C: High-density lipoprotein cholesterol
LDL-C: Low-density lipoprotein cholesterol
CRP: C-reactive protein
SNUH: Seoul National University Hospital
MCA: Middle cerebral artery
ICA: Internal carotid artery
PCA: Posterior cerebral artery
BA: Basilar artery
CT: Computed tomography
MRI: Magnetic resonance image
mRS: Modified Rankin scale
TOAST: Trial of ORG 10,172 in Acute Stroke Treatment
mTICI: Modified thrombolysis in cerebral infarction
IQR: Interquartile range
